# Respiratory variations in aortic blood flow velocity and inferior vena cava diameter as predictors of fluid responsiveness in mechanically ventilated children using transthoracic echocardiography in a pediatric PICU

**DOI:** 10.1186/cc14261

**Published:** 2015-03-16

**Authors:** K El Halimi, M Negadi, H Bouguetof, L Zemour, D Boumendil, Z Chentouf Mentouri

**Affiliations:** 1 University Ahmed Benbella Oran 1, Oran, Algeria

## Introduction

Volume expansion remains the first treatment step for most children with acute circulatory failure in order to assess blood volume status. In this way, dynamic echocardiographic parameters have been proposed in mechanically ventilated children [1,2], using the heart-lung interactions. This study aimed to investigate whether respiratory variations of aortic blood flow velocity (DELTA Vpeak ao) and inferior vena cava diameter (DELTA IVC) by transthoracic echocardiography (TTE) could accurately predict fluid responsiveness in ventilated children.

## Methods

A prospective observational and interventional study conducted in a pediatric ICU investigated 40 mechanically ventilated children with preserved left ventricular (LV) function using TTE. Each patient had tachycardia, hypotension, oliguria, delayed capillary refilling or hemodynamic instability despite vasopressor drugs. Intervention: standardized volume expansion (VE).

## Results

The VE-induced increase in LV stroke volume was ≥10% in 28 patients (responders) and <10% in 12 patients (nonresponders). Before VE, the DELTA Vpeak ao and DELTA IVC in responders was respectively higher than that in nonresponders (18.75% (12 to 32) vs. 13.5% (6 to 16) and 31% (18 to 57) vs. 17.5% (14 to 25)). The prediction of fluid responsiveness was higher with DELTA Vpeak ao (ROC curve area 0.894 (95% CI = 0.756 to 0.969) *P *= 0.0001) and DELTA IVC (ROC curve area 0.869 (95% CI = 0.717 to 0.957), *P *= 0.0001). The best cutoff value for DELTA Vpeak ao was 16% with sensitivity and specificity predictive values of 71.6% and 83.3%, respectively, and DELTA IVC was 20% with sensitivity and specificity predictive values of 88.5% and 90.9%, respectively.

## Conclusion

In this study, DELTA Vpeak and DELTA IVC were appropriate variables to predict fluid responsiveness by TTE in ventilated children.
